# Comparison of blind intubation with different supraglottic airway devices by inexperienced physicians in several airway scenarios: a manikin study

**DOI:** 10.1007/s00431-019-03345-4

**Published:** 2019-03-22

**Authors:** Andrzej Bielski, Jacek Smereka, Marcin Madziala, Dawid Golik, Lukasz Szarpak

**Affiliations:** 1 Polish Society of Disaster Medicine, Warsaw, Poland; 2 Department of Emergency Medical Service, Wroclaw Medical University, Wroclaw, Poland; 3 Lazarski University, 43 Swieradowska Str., 02-662 Warsaw, Poland

**Keywords:** Endotracheal intubation, Child, Emergency medicine, Difficult airway, Medial simulation

## Abstract

Endotracheal intubation is the gold standard for airway management. Supraglottic airway devices (SADs) are useful in airway abnormalities. SAD blind intubation enables airway management with better ventilation and a reduced risk of gastric content aspiration. The aim was to compare various SADs in blind intubation performed by inexperienced physicians in several pediatric airway scenarios. One hundred sixteen physicians with no previous experience with SAD performed blind endotracheal intubations with (1) iGEL, (2) Air-Q intubating laryngeal airway, and (3) Ambu AuraGain disposable laryngeal mask in a pediatric manikin in three airway scenarios: (A) normal airway without chest compressions, (B) normal airway with continuous chest compressions with the CORPULS CPR system, and (C) difficult airway with continuous chest compressions with the CORPULS CPR system. Intubation tube with 5.0 internal diameter was used for all blind intubation attempts. First intubation success rate, median time to SAD placement, time to endotracheal intubation with SAD, and ease to perform the intubation were investigated in this study. All these parameters were better or non-inferior for iGEL in all investigated scenarios.

*Conclusion*: Our manikin study demonstrated that iGEL was the most effective device for blind intubation by inexperienced physicians in different pediatric airway scenarios.

**Table:** 

**What is Known:** *• For pediatric resuscitation, bag-mask ventilation is the first-line method for airway control and ventilation.* *• Endotracheal intubation is considered by many scientific societies the gold standard for airway management.* *• Supraglottic airway devices are particularly useful when bag-mask ventilation is difficult or impossible but can be also used for blind intubation.*	
**What is New:** *• The iGEL laryngeal mask turns out the most effective device for blind intubation by inexperienced physicians in different pediatric airway scenarios.* *• It may be a reasonable first emergency blind intubation technique for inexperienced physicians in pediatric patients in normal airway with and without continuous chest compressions, as well as in difficult airway with continuous chest compressions.*

## Introduction

For pediatric resuscitation, bag-mask ventilation remains the recommended first-line method for airway control and ventilation [23]. However, intubation is among the most important procedures related to prognosis in severe cases [24]. Endotracheal intubation is considered by many scientific societies the gold standard for airway management [9, 23]. The need to quickly ensure airway patency becomes particularly important in pediatric patients, in whom the inability to maintain airway patency and progressive hypoxia are the primary causes for cardiac arrest.

Endotracheal intubation in pediatric patients is more challenging for medical personnel with greater risk of technical problems and complications. The success rate is influenced by several factors, including the intubator’s experience. Failed attempts can pose a risk for airway edema, hypoxia, and bleeding [17].

Supraglottic airway devices (SADs) are particularly useful in airway abnormalities when bag-mask ventilation is difficult or impossible [31]. It should be taken into account that SADs do not totally protect the airway from aspiration, and therefore blind endotracheal intubation with a SAD can offer some advantages compared with SAD. The airway anatomy in pediatric patients differs that in adults; pediatric intubation requires special training and ongoing experience. The European Resuscitation Council 2015 guidelines recommend that in pediatric cardiac arrest, endotracheal intubation should be performed by an experienced and trained practitioner [23].

Blind intubation with SAD is a technique for endotracheal tube insertion through the airway channel of the SAD enabling airway management with better ventilation and a reduced risk of gastric content aspiration [4, 19, 20]. In clinical conditions, fiberoptic techniques can be used to check the correct position and to allow endotracheal intubation with visual control [34]. For emergency situations, though, especially in the prehospital setting or with novice physicians, these are difficult or impossible to obtain.

The authors assumed that unexperienced physician can attempt endotracheal intubation through the established SAD to protect the airway, which is particularly useful in long-lasting resuscitation efforts where there is a lack of availability of experienced medical personnel and reduces the risk of gastric content aspiration.

The aim of the study was to compare the application of various SADs for blind intubation performed by inexperienced physicians in different pediatric airway scenarios.

## Methods

### Ethical approval and informed consent

This study was designed as a prospective, randomized crossover observational trial, with written informed consent regarding the study purpose obtained from all participants. The Institutional Review Board of the International Institute of Rescue Research and Education approved the study protocol (approval No. 31.06.2017.IRB).

### Participants

The study involved 120 nonanesthetic and nonemergency physicians participating in emergency medicine training at Wroclaw Medical University. It was conducted between December 2017 and April 2018; 116 physicians completed it. No participant had previously used or had been formally instructed in SAD application in experimental or clinical situations. Those experienced with SADs were excluded. Among the exclusion criteria, there was also wrist injury or spinal injury during the 3 months preceding the study.

### Devices

To standardize the participants’ knowledge and skills in airway management with SADs, as well as in blind intubation with SADs as a guide for the endotracheal tube, all participants underwent a 30-min theoretical training on pediatric airway anatomy, physiology, and pathophysiology of breathing, and methods of airway management, including SADs and blind intubation. At the end of the training, the instructor demonstrated the correct use of the tested SADs and the implementation of blind intubation with their use. Blind intubation consists of two parts, the correct placement of SAD and the subsequent introducing of the endotracheal tube through SAD.

Then, the study participants had 10 attempts to practice SADs and blind intubation on an adult airway model; the AT Kelly Torso manikin (Laerdal, Stavanger, Norway) was used.

**Fig. 1:**
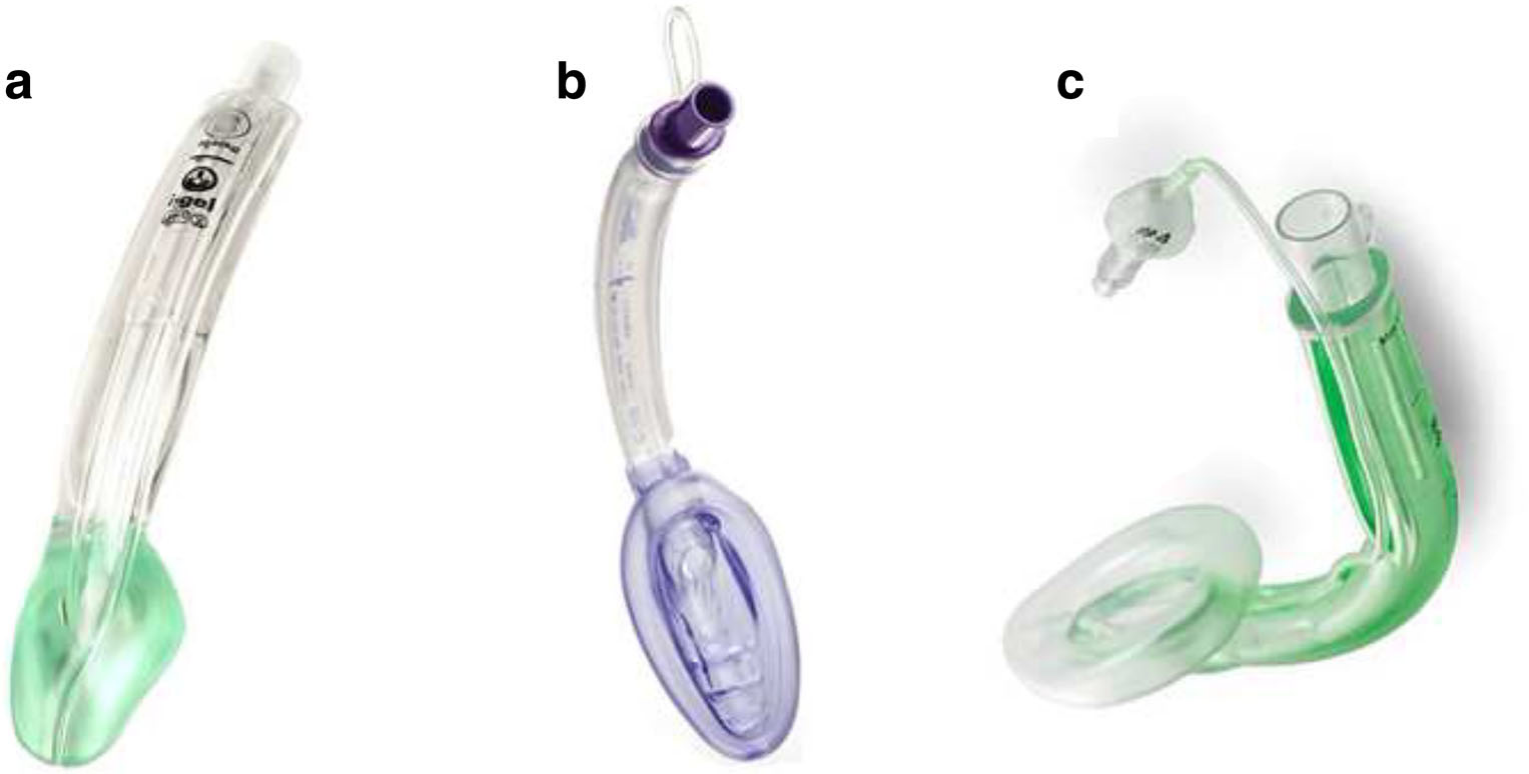
Supraglottic airway devices used in the study: (A) iGEL, (B) Air-Q, and (C) AMBU

The study included three SADs:

1. The iGEL™ (iGEL; Intersurgical, Berkshire, UK), a type of laryngeal mask airway device. The cuff is constructed of medical grade thermoplastic elastomer, and there is no need to inflate it or adjust its pressure [33].
2. The Air-Q® intubating laryngeal airway (Air-Q; ILA™, Cookgas® LLC, Mercury Medical, Clearwater, FL, USA), an aid for airway maintenance and a conduit for tracheal intubation during general anesthesia [1].
3. The Ambu® AuraGain™ disposable laryngeal mask, size 2½ (Ambu; Ambu A/S, Ballerup, Denmark), a third-generation laryngeal mask, addressing 3 fundamental airway management needs by integrating gastric access and intubation capability in an anatomically curved single-use device, facilitating rapid safe airway establishment [22] (Fig. 1).

The intubation tube with 5.0 internal diameter was used for all blind intubation attempts. Both the endotracheal tube and the SAD airway channel were moisturized with a lubricant. Additionally, to confirm the blind intubation effectiveness, the participants attempted to ventilate the patient’s lungs with a self-inflating bag.

### Study protocol

To simulate a 5-year-old requiring immediate airway protection, we used the Pediatric HAL® S3005 simulator (Gaumard® Scientific, Miami, FL, USA).

The physicians participated in three airway scenarios:

1. Scenario A: normal airway without chest compressions during the intubation attempt.
2. Scenario B: normal airway with continuous chest compressions during the intubation attempt. The CORPULS CPR chest compression system (GS Elektromedizinische Geräte G. Stemple GmbH, Kaufering, Germany), also dedicated to pediatric patients, was applied. Chest compressions were performed continuously, 100 compressions per minute, each with the depth of 5 cm.
3. Scenario C: difficult airway with continuous chest compressions. Here, the CORPULS CPR system was also applied. Tongue swelling was obtained with simulator control software to generate a Mallampati grade 3. The intubation difficulty was each time evaluated by an experienced anesthesiologist.

For each test scenario, the patient was placed on the floor on a hard surface in a bright room to simulate emergency settings in pre-hospital care.

**Fig. 2:**
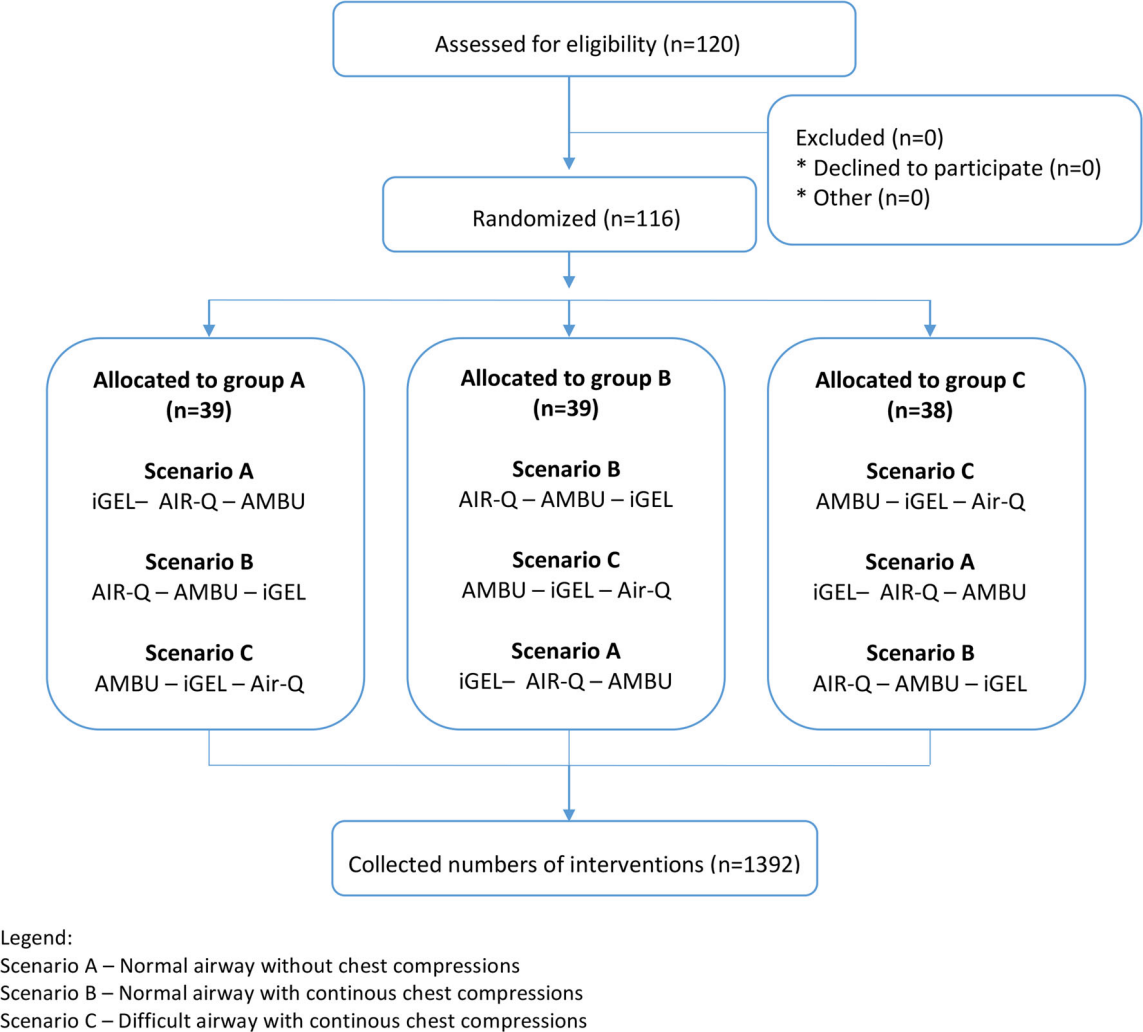
Randomization flow chart

Each participant performed airway management for each tested SAD and then blind intubation with particular SADs as conducts for endotracheal tube. There were maximally 3 attempts with each device in each scenario. The order of both the participants and airway management methods were random; the Research Randomizer software (randomizer.org) was used. The detailed randomization procedure is shown in Fig. 2.

### Measurement definitions

The primary outcome was the rate of intubation success, as recorded by the simulator indicators. Ineffective intubation was defined as intubation lasting over 120 s or intubation of the esophagus during three consecutive attempts. Both the first intubation efficacy and the overall efficacy were assessed. Secondary outcomes were time parameters and the ease of use. The time to successful SAD placement was measured from when the participant touched the assigned SAD until the device was placed in the airway and secured by the cuff inflation. Additionally, the time to blind intubation was measured, and defined as the time between the operator’s picking up the SAD and establishing manual ventilation via the endotracheal tube. To provide their subjective opinion about each intubation method difficulty, the participants rated it on a visual analog scale (VAS), from 1 (extremely easy) to 100 (extremely difficult) points.

### Sample size and statistical analysis

The sample size was calculated with G*Power 3.1 with a two-tailed *t* test (Cohen’s *d* = 0.8, alpha error = 0.05, power = 0.95). With the minimum of 87 participants necessary, 116 physicians were included to compensate for potential doubts.

The data were recorded in the previously prepared study data form, and the Statistica 13.0EN (StatSoft, Tulsa, OK, USA) was used for the analysis. Normal distribution of numerical variables was tested with the Kolmogorov-Smirnov test. To detect differences in the intubation success rate, the McNemar test was applied. The two-sided Wilcoxon signed-rank test allowed to compare the procedure time. The participants’ subjective opinions (VAS score) were compared with the Stuart-Maxwell test. Data were presented as medians (interquartile ranges, IQR) or percentages. The *p* value < 0.05 was considered significant.

## Results

The study involved 116 physicians (71 females, 61.2%) with median age 33 (IQR, 28–40) years and median work experience 7 (IQR, 4–11) years. All participants declared knowledge of endotracheal intubation with a laryngoscope with Miller and Macintosh blades; however, none had experience in SAD airway management.

### Scenario A: normal airway without chest compressions

The first intubation success rate varied with distinct devices and amounted to 85.3% vs. 71.6% vs. 76.7% (iGEL, Air-Q, and Ambu, respectively; Table 1). There was a statistically significant difference between iGEL and Air-Q (*p* = 0.011) and between iGEL and Ambu (*p* = 0.043). The total intubation efficiency was 100% with iGEL and Ambu, and 96.6% for Air-Q (*p* = 0.046).

**Table 1:** Study outcomes by supraglottic airway devices with unexperienced physician (*N* = 116)

Outcome	iGEL	Air-Q	AMBU	*p* value
Scenario A: normal airway without chest compression				
Success of first intubation attempt [%]	99 (85.3%)	83 (71.6%)	89 (76.7%)	iGEL vs. Air-Q = 0.011 iGEL vs. AMBU = 0.043 Others: NS
Overall intubation success rate [%]	116 (100%)	112 (96.6%)	116 (100%)	iGEL vs. = 0.046 AMBU vs. = 0.046 Others: NS
Time to supraglottic airway device placement [s]	9.5 [8–14]	16.5 [14–21]	15 [13–18.5]	iGEL vs. Air-Q = 0.002 iGEL vs. AMBU = 0.013 Others: NS
Time to endotracheal intubation [s]	18 [15–20]	28 [18–33]	27 [17–31]	iGEL vs. Air-Q = 0.008 iGEL vs. AMBU = 0.016 Air-Q vs. AMBU = 0.048
Ease of use, 1–100 scale	29 [22–35]	39 [28–41]	36 [31–36]	iGEL vs. Air-Q = 0.023 iGEL vs. AMBU = 0.021 Air-Q vs. AMBU = 0.041
Scenario B: normal airway with continuous chest compression				
Success of first intubation attempt [%]	93 (80.2%)	80 (68.9%)	81 (69.8%)	iGEL vs. Air-Q < 0.001 iGEL vs. AMBU <0.001 Others: NS
Overall intubation success rate [%]	116 (100%)	101 87.1%)	116 (100%)	iGEL vs. Air-Q = 0.001 AMBU vs. Air-Q = 0.001 Others: NS
Time to supraglottic airway device placement [s]	9 [8–15]	17 [15–23]	15.5 [14–20]	iGEL vs. Air-Q = 0.001 iGEL vs. AMBU = 0.003 Air-Q vs. AMBU = 0.038
Time to endotracheal intubation [s]	18 [15–20.5]	28 [23–39.5]	27 [17–32]	iGEL vs. Air-Q < 0.001 iGEL vs. AMBU <0.001 AMBU vs. Air-Q = 0.045
Ease of use, 1–100 scale	29 [24–36]	41 [34–47]	37 [31–39]s	iGEL vs. Air-Q = 0.003 iGEL vs. AMBU = 0.011 AirQ vs. AMBU = 0.044
Scenario C: difficult airway with continuous chest compression				
Success of first intubation attempt [%]	89 (76.7%)	71 (61.2%)	82 (70.7%)	iGEL vs. Air-Q = 0.007 iGEL vs. AMBU = 0.022 Air-Q vs. AMBU = 0.016
Overall intubation success rate [%]	104 (89.7%)	95 (81.9%)	100 (86.2%)	iGEL vs. Air-Q = 0.011 Air-Q vs. AMBU = 0.015 Others: NS
Time to supraglottic airway device placement [s]	10.5 [9–15]	18.5 [15–24]	16 [14–21.5]	iGEL vs. Air-Q = 0.001 iGEL vs. AMBU = 0.004 Air-Q vs. AMBU = 0.038
Time to endotracheal intubation [s]	19 [17–25]	31 [25.5–43]	28 [22–34.5]	iGEL vs. Air-Q < 0.001 iGEL vs. AMBU = 0.019 Air-Q vs. AMBU = 0.033
Ease of use, 1–100 scale	30 [25–38]	41 [35–49]	37 [31–40]	iGEL vs. Air-Q = 0.009 iGEL vs. AMBU = 0.015 Air-Q vs. AMBU = 0.039

**Fig. 3:**
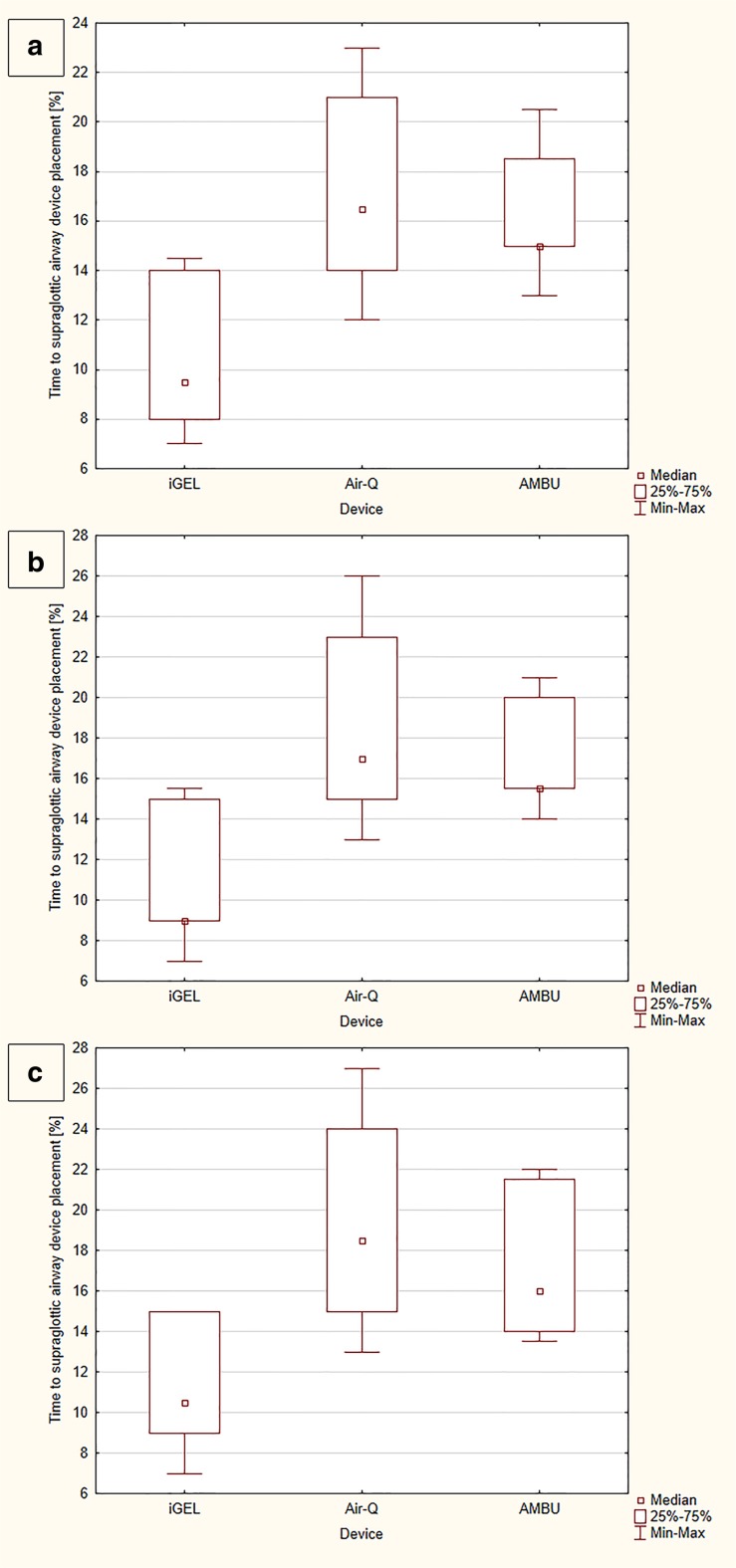
Time to supraglottic airway device placement during study scenarios

**Fig. 4:**
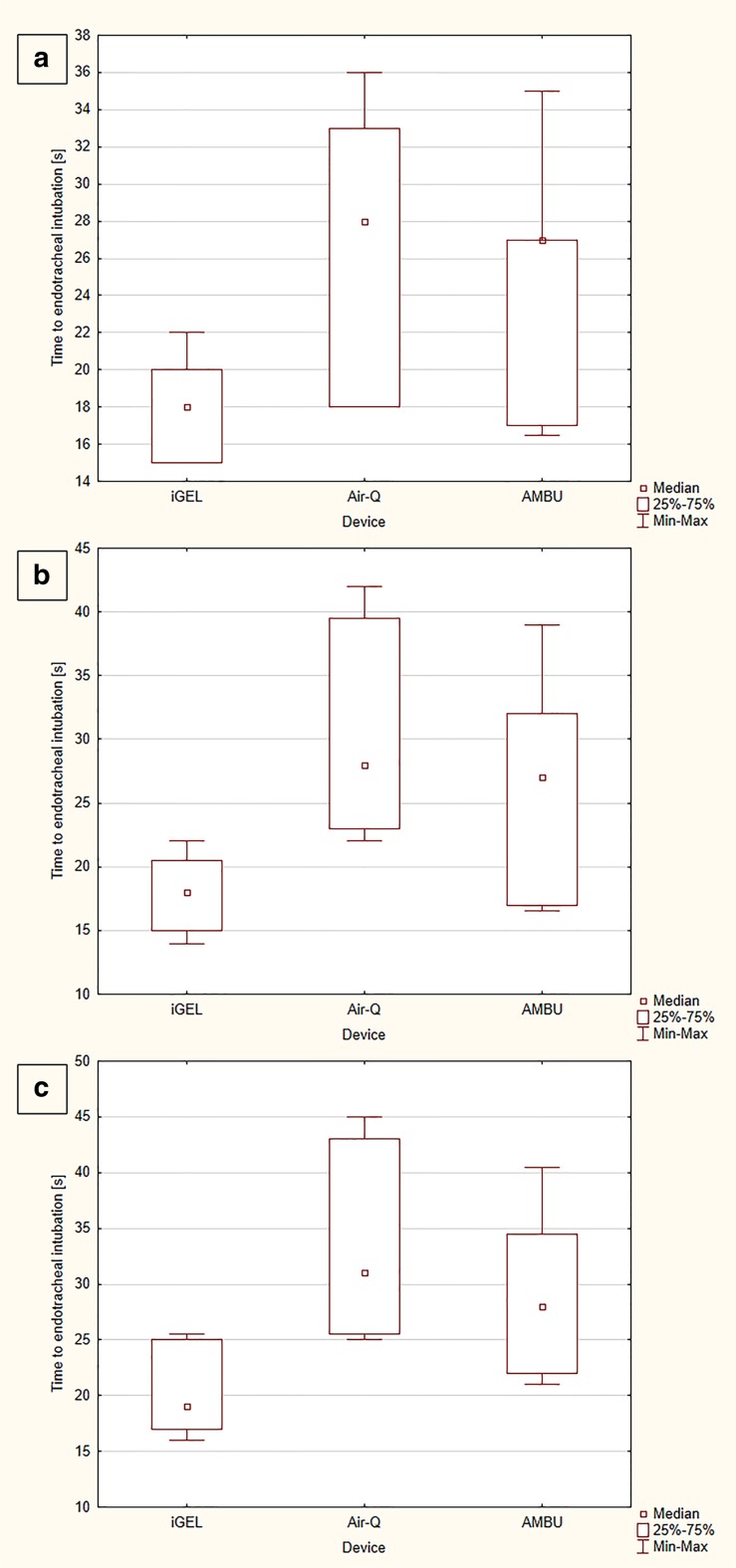
Time to endotracheal intubation during study scenarios

The median time to SAD placement equaled 9.5 (IQR, 8–14) seconds for iGEL, which was statistically significantly shorter than with Air-Q (16.5 [IQR, 14–21] seconds) (*p* = 0.002) and with Ambu (15 [IQR, 13–18.5] seconds) (*p* = 0.013; Fig. 3a). The time to endotracheal intubation with iGEL, Air-Q, and Ambu was 18 (IQR, 15–20) vs. 28 (IQR, 18–33) vs. 27 (IQR, 17–31) seconds, respectively (Fig. 4a).

**Fig. 5:**
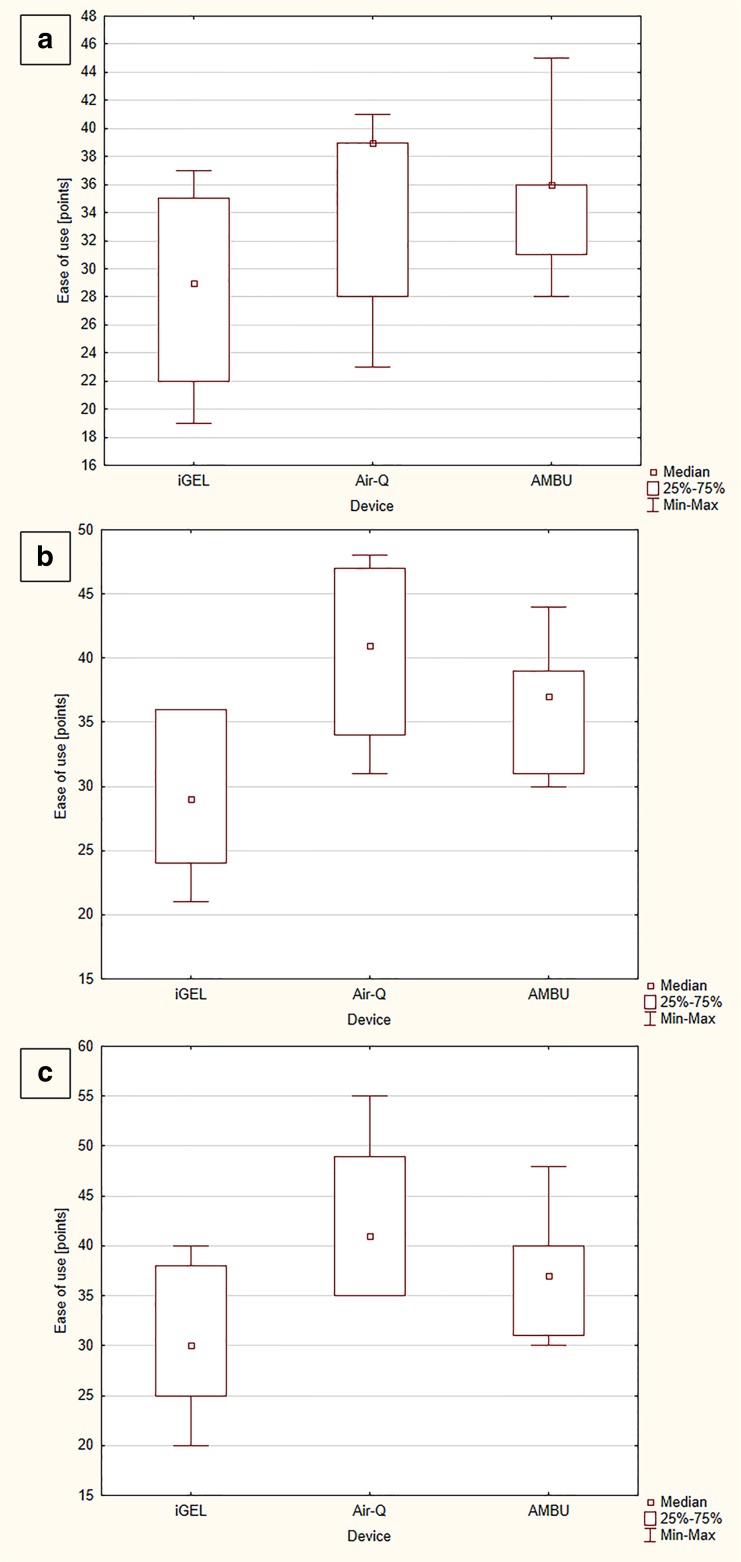
Ease of use scale

The ease to perform blind intubation with iGEL equaled 29 (IQR, 22–35) points, which was statistically significantly better than for Air-Q (39 [IQR, 28–41] points) (*p* = 0.023) and for Ambu (36 [IQR, 31–36] points) (*p* = 0.021). A statistically significant difference was also observed between Air-Q and Ambu (*p* = 0.041; Fig. 5a).

### Scenario B: normal airway with continuous chest compressions

The first blind intubation effectiveness with iGEL, Air-Q, and Ambu varied and equaled 80.2% vs. 68.9% vs. 69.8%, respectively (Table 1). A statistically significant difference was observed between iGEL and Air-Q (*p* < 0.001) and between iGEL and Ambu (*p* = 0.001). The total intubation efficiency with iGEL, Air-Q, and Ambu was 100%, 87.1%, and 100%, respectively.

The median time to SAD placement was 9 (IQR, 8–15) seconds for iGEL, 17 (IQR, 15–23) seconds for Air-Q, and 15.5 (IQR, 14–20) seconds for Ambu (Fig. 3b). There were statistically significant differences between iGEL and Air-Q (*p* = 0.001), iGEL and Ambu (*p* = 0.003), and Air-Q and Ambu (*p* = 0.038). In turn, the median time to endotracheal intubation with iGEL equaled 18 (IQR, 15–20.5) seconds and was statistically significantly shorter than for Air-Q (28 [IQR, 23–39.5] seconds) (*p* < 0.001) and for Ambu (27 [IQR, 17–32] seconds) (*p* < 0.001; Fig. 4b).

The ease of performing blind intubation was varied: 29 (IQR, 24–36) points for iGEL, 41 (IQR, 34–47) points for Air-Q, and 37 (IQR, 31–39) points for Ambu (Fig. 5b). Applying iGEL was the simplest method (*p* < 0.02).

### Scenario C: difficult airway with continuous chest compressions

The first intubation success rate was 76.7% for iGEL, 61.2% for Air-Q, and 70.7% for Ambu (Table 1). Statistically significant differences were noted between iGEL and Air-Q (*p* = 0.007), iGEL and Ambu (*p* = 0.022), and Air-Q and Ambu (*p* = 0.016). The overall blind intubation effectiveness in the case of iGEL, Air-Q, and Ambu equaled 89.7%, 81.9%, and 86.2%, respectively. Statistically significant differences were found between iGEL and Air-Q (*p* = 0.011) and between Air-Q and Ambu (*p* = 0.015).

The time to SAD placement amounted to 10.5 (IQR, 9–15) seconds for iGEL, 18.5 (IQR, 15–24) seconds for Air-Q, and 16 (IQR, 14–21.5) seconds for Ambu (Fig. 3c). There was a statistically significant difference between iGEL and Air-Q (*p* < 0.001), iGEL and Ambu (*p* = 0.004), and Air-Q and Ambu (*p* = 0.038).

The median time to blind intubation with iGEL equaled 19 (IQR, 17–25) seconds, which was statistically significantly shorter compared with Air-Q (31 [IQR, 25.5–43] seconds) (*p* < 0.001) and Ambu (28 [IQR, 22–34.5] seconds) (*p* = 0.019; Fig. 4c).

The intubation ease amounted to 30 (IQR, 25–38) points for iGEL, 41 (IQR, 35–49) points for Air-Q, and 37 (IQR, 31–40) points for Ambu. There was a statistically significant difference between iGEL and Air-Q (*p* = 0.009), iGEL and Ambu (*p* = 0.015), and Air-Q and Ambu (*p* = 0.039; Fig. 5c).

## Discussion

The presented study evaluated the effectiveness of blind intubation with different SADs as a conduct for endotracheal tube in several pediatric airway scenarios. The iGEL laryngeal mask turned out the most effective. During airway management in pediatric patients, the anatomical and physiological features of pediatric patients, described in the introduction, should be taken into account. Hence, a lower first intubation efficacy may result in this group. Among 12 inexperienced users in a study by Balaban et al., the time to endotracheal intubation with the Miller laryngoscope was 72 ± 45 s compared with 72 ± 45 s for the Macintosh laryngoscope [2]. In a study by Szarpak et al. [28], paramedics performed endotracheal intubation in a pediatric manikin under normal airway conditions within 24.3 s while maintaining 100% efficacy using a Macintosh laryngoscope. These differences can be explained by the degree of intubation training in the studied groups.

Paramedics are trained to perform direct laryngoscopy throughout a 3-year course to be able to perform the procedure in emergency settings, when time pressure is extremely stressful and could affect less experienced personnel.

Numerous studies indicate that in pediatric patients over 2 years of age, laryngoscopes with both Miller and Macintosh blade can be used with comparable effectiveness [36]. In turn, Eisenberg et al. [10] showed that in a pediatric emergency department, the first intubation effectiveness with the use of direct laryngoscopy was only 71%.

Burns et al. [7], in a study regarding first-look success in emergency pediatric intubation by a physician-staffed helicopter emergency medical service, demonstrated registrars achieving a first-look success to rate 26 of 26 (100%), consultants 16 of 17 (94%), and paramedics 33 of 39 (85%). Studies performed in adults intubated in emergency departments also indicate inadequate first intubation efficacy [13, 14]. It is important to perform endotracheal intubation during the first attempt because, as proved by Benumof [3], with more than 3 intubation attempts, each subsequent one is associated with the risk of soft tissue bleeding and airway edema, potentially leading to a situation determined by the Difficult Airway Society as “cannot intubate, cannot ventilate” [8], with the solution of performing conjugation or tracheostomy [25].

Therefore, it is crucial to search for methods of endotracheal intubation alternative to direct laryngoscopy. These include SADs, with a possibility to perform blind intubation by using the SAD ventilation channel as a guide for the endotracheal tube [16, 21]. In the presented study, the first attempt effectiveness with all the tested devices exceeded 71%, being the highest with the iGEL mask (85.3%). For iGEL and Ambu, the total blind intubation success achieved 100%. Also, the intubation time with the tested devices was shorter than that shown by Balaban [2]. Jagannathan [12] demonstrated that the time of fibreoptic bronchoscopy-guided intubation in pediatric patients via iGEL and Air-Q was 55.9 (48.5–81.8) seconds vs. 62.5 (47.9–77) seconds. This longer intubation time is dictated by the differences in the intubation procedure. The time of intubation is of key importance as children have a high metabolic rate to the total body surface, leading to rapid hypoxia [11]. In the endotracheal intubation settings, an important parameter affecting resuscitation effectiveness is the minimization of chest compression interruptions. Airway management with direct laryngoscopy should be thus performed during uninterrupted chest compressions, which is associated with a significant reduction in intubation effectiveness and a longer procedure [5, 6, 18].

The median intubation time analysis in the own study revealed no increase in duration between intubation with and without chest compressions in relation to the tested devices. The first intubation effectiveness decreased slightly, but it was above 68%, although 80.2% with iGEL. A lower effectiveness of direct laryngoscopy performed by experienced paramedics in simulated cardiopulmonary resuscitation pediatric conditions was shown by Szarpak et al. [30]: the intubation effectiveness with Miller laryngoscope was 77.5%.

In emergency medicine settings or emergency airway management, each patient should be treated as one with difficult airways. Thus, one should be prepared for difficulties in performing endotracheal intubation with direct laryngoscopy [32].

With SADs, this problem is minimized. In our study, the effectiveness of first blind intubation was 76.7% with iGEL, 70.7% with Ambu, and 61.2% with Air-Q. A comparable efficacy (68.2%) of the first intubation attempt with pediatric difficult airway in a scenario with chest compressions performed by paramedics was observed by Szarpak et al. [29].

In all scenarios of the presented study, iGEL turned out the easiest method of blind intubation. Kim et al. [15] showed that iGEL had an easier insertion and better sealing function than Air-Q in children requiring general anesthesia. The simplicity of the procedure and the shorter learning curve with SADs than with direct laryngoscopy may be influenced by more effective airway protection, especially in adverse conditions, undoubtedly including cardiopulmonary resuscitation [26], or patients with difficult airways [4, 20].

The study has some limitations. Firstly, it was performed in simulation conditions, not in real emergency situations. However, the usage of an advanced simulator was dictated by the fact that cross-over randomized studies in cardiopulmonary resuscitation are unethical [27, 35], and medical simulators allow for full standardization of medical procedure conditions without any potential harm to patients. Secondly, the study was limited to physicians. It is, however, the professional group relatively often facing the need to perform airway management in pediatric patients. With negligible or complete lack of experience in direct laryngoscopy, SAD blind intubation may constitute an alternative to endotracheal intubation.

Our aim was not to suggest that it is the physician’s duty to try to perform blind intubation but to emphasize that SAD which has an established position in the field of airway management can be also used to perform blind intubation in certain circumstances.

Our study also has several strengths. These include the cross-over randomized design, the usage of three modern SADs and one of the world’s most advanced pediatric simulators, and a large size of the study group.

## Conclusions

Considering the first intubation success rate, median time to SAD placement, and ease to perform blind intubation, the manikin study demonstrated iGEL to be the most effective device for blind intubation by inexperienced physicians in different pediatric airway scenarios. The iGEL laryngeal mask may be a reasonable first emergency blind intubation technique for inexperienced physicians in pediatric patients with normal airway with and without continuous chest compressions, as well as with difficult airway with continuous chest compressions.

## Abbreviations

AMBU: The Ambu® AuraGain™ Disposable Laryngeal Mask
CPR: Cardiopulmonary resuscitation
iGEL: iGEL laryngeal mask
IQR: Interquartile range
IRB: Institutional review board
SAD: Supraglottic airway device
VAS: Visual analog scale
